# The FAST Pump, a low-cost, easy to fabricate, SLA-3D-printed peristaltic pump for multi-channel systems in any lab

**DOI:** 10.1016/j.ohx.2020.e00115

**Published:** 2020-06-07

**Authors:** Alexander Jönsson, Arianna Toppi, Martin Dufva

**Affiliations:** DTU Health Tech, Technical University of Denmark, Kgs Lyngby, Denmark

**Keywords:** Open source hardware, Open hardware, 3D-printing, Stereolithography, SLA, DLP, Off the shelf, Ease-of-use, DIY

## Abstract

With the increasing interest in high throughput screening and parallel assays, laboratories around the world inevitably find themselves in need of driving a multitude of fluid lines to facilitate their large scale studies. The comparatively low cost and no-fluid-contact design of peristaltic pumps make them the go-to systems for such ventures, but using commercially available pumping systems this still becomes a costly endeavor at typically $250-$1000 per pump line. Here we have developed an alternative, a peristaltic pump that can be fabricated in most research laboratories using 3D-printing and readily available off-the-shelf parts. The pump features 8 parallel channels with linear ranges spanning from 0.7 µL/min to 6 mL/min. The pump can be fabricated and assembled by anyone with access to a 3D-printer at a cost of less than $45 per channel and is driven by a stepper motor that connects directly to any computer. This device has the potential to be disruptive in areas such as drug screening and assay development, as well as lab-on-a-chip applications and cell cultivation, where it significantly reduces hardware expenses and allows for construction of more comprehensive fluidic systems at a fraction of current costs.

## Graphical abstract

**Figure:**
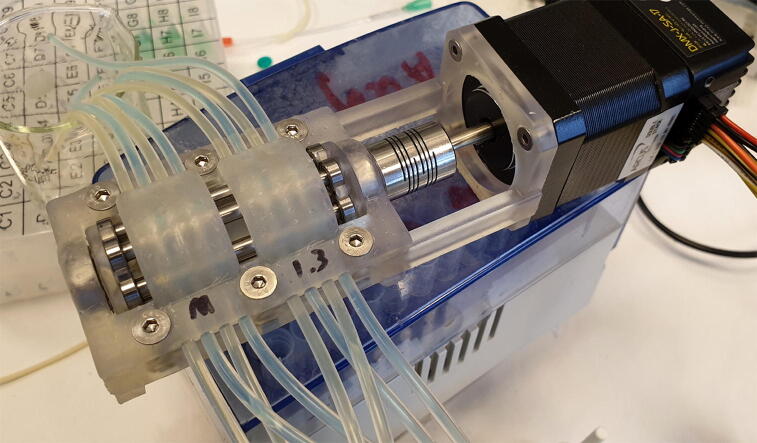

Specifications table

**Table:** 

Hardware name	*FAST Pump*
Subject area	• Educational Tools and Open Source Alternatives to Existing Infrastructure
Hardware type	• Biological sample handling and preparation • Mechanical engineering and materials science
Open Source License	*GNU General Public License v. 3*
Cost of Hardware	*US $362.37*
Source File Repository	*https://doi.org/10.17605/OSF.IO/3R7H4*

## 1 Hardware in context

The use of microfluidics and Lab-on-a-chip based systems is by now a well-established part of the research workflow in fields such as analytical chemistry, drug development, cell biology, tissue engineering and point of care diagnostics [1], [2], [3], [4], [5], [6], [7], [8]. These miniaturized systems have several benefits over their larger, conventional counterparts, such as lower price, smaller sample volumes, less use of reagents and an overall smaller size [9]. Together, these benefits make it possible to run multiplexed processes such as parallel assays or cell cultures [10], [11], [12], [13], but this increased throughput also comes with its own hurdles in supplying controlled fluid flow. There are a number of possible solutions, with micropumps included on or off the actual device [14], [15], [16], [17], [18]. While some offer great versatility for highly multiplexed systems[19], they all typically rely on highly specialized fabrication methods not easily accessible to researchers outside the field of microfabrication [19], [20], [21] or advanced machining [22]. In many cases this means that the easiest, and indeed only, solution is to use stand-alone commercial pumps such as syringe pumps, pressure pumps or peristaltic pumps. While these offer accurate control of the fluid flow the price can be a major hurdle, with even peristaltic pumps, the cheapest of the three types, typically being priced in the hundreds of dollars to over $1000 per pumped channel. The physical size of the pumps themselves also rules out the possibility of truly compact systems.

The ever increasing popularity of 3D-printing provides a possible solution to these issues as it is now possible to fabricate complex parts at the push of a button. This allows researchers without previous training in advanced machining, nor access to a mechanical workshop, to produce functional devices to be used in the laboratory [23], [24], [25], [26], [27].

Fused Filament Fabrication (FFF), also known as Fused Deposition Modeling (FDM), is perhaps the most well-known 3D-printing process for consumer use, with its low cost and straightforward fabrication workflow [28]. With FDM 3D-printing it is possible to quite rapidly, and with minimal post processing, produce polymer parts that can be assembled into functional equipment and peristaltic pumps for laboratory use fabricated this way have indeed been presented [29], [30]. Drawbacks in the form of comparatively low feature resolution and weak mechanical properties [31], both related to the layer by layer deposition method, could be the reason why, despite the prevalence of FDM printers, few pumps are seen in literature. The related process of Inkjet 3D-printing is typically not available to the average consumer, nor most research laboratories, due to the high price, but can provide higher resolution [32]. Pump designs printed using this technology will show a similar layer-by-layer structure to FDM printed parts, including poor layer-to-layer adhesion [33], but has nonetheless been presented in a number of publications [34], [35], [36]. Common for all published, 3D-printed, peristaltic pumps at the time of writing, with the exception of the highly specialized design by Nightingale et al. [36], only feature one pumped channel and thus lend themselves poorly to parallel setups.

In contrast to both FDM and Inkjet printing the two related processes of Stereolithography (SLA) and Digital Light Processing (DLP) produce monolithic printed parts without distinct layers [31], resulting in mechanical properties closer those of injection molded parts and with high feature resolution. This makes these “resin”-based printers more suitable to fabricating highly reproducible parts with high demand for dimensional tolerances, as is the case with peristaltic pumps. In recent years these printers have become more available to the average consumer, with DLP models now selling for less than $350, like the Elegoo Mars (www.elegoo.com) and Sparkmaker FHD (www.sparkmaker3d.com). SLA printers provide higher resolution, and better surface finish, and many models are priced within reach for any research laboratory or high end consumer. Examples include the Peopoly Moai (www.peopoly.com) and Formlabs Form 2/3 (www.formlabs.com).

In an attempt to provide the scientific community with an affordable alternative to expensive, commercial, pumps we here present a low-cost, in-house fabricated, peristaltic pump. The pump is constructed using only 3D-printed parts, printed on a Form 2 3D-printer, and commercially available “off the shelf” components. Specifically this paper describes the design, fabrication, assembly and operation of the FAST Pump, a low-cost, no-required-skill, 3D-printed peristaltic pump for multi-channel systems.

## 2 Hardware description

The FAST Pump is a small scale, peristaltic pump built on the same principle as many commercial pumps, featuring multiple free-spinning rollers on a central shaft. It is a horizontal, free standing, pump featuring six all-metal rollers. The design includes the main pump body, three lid variants, and the central shaft. It also includes a jig needed for assembly of the rollers. Based around open-source and accessibility, all parts can be 3D-printed and assembled using a minimal variety of tools. The pump is driven by a common NEMA 17 stepper motor, with integrated driver and controller, and controlled by the manufacturer’s software. The printing time for a complete set of parts is about 14 h, using a Form 2 3D-printer at 50 µm layer height, and assembly can be done in under 1 h.

The low cost FAST Pump can be built for less than $400 in material costs and is designed with ease-of-use in mind. For this reason a high quality integrated stepper motor is used even though its price represent almost ¾ of the pumps total cost. This essentially plug-and-play configuration can easily be replaced with a bare-bone NEMA 17 stepper motor and separate controller and driver for a fraction of the cost. In this case it should be possible to construct the FAST pump at a total cost of less than $150.

The FAST pump differs from other open source pumps in three main ways; i) the number of parallel channels, ii) it only uses 3D-printed or off the shelf standard parts easily found on-line iii) it can be assembled in a short time using minimal tools. The pump also differs from commercial pumps in three main ways; i) the small size at about 1/6th of the footprint of a commercial 8-channel pump, ii) the price at about 1/10th of an average priced 8-channel pump, iii) modularity, as the FAST Pump can be modified to fit into e.g. integrated fluidics platforms. Together these attributes make the FAST Pump ideal for research environments where a high number of parallel channels is needed and were integration is of concern. The small size also make it an attractive alternative for day-to-day pumping needs.

The FAST Pump

• Is a low cost alternative to commercial peristaltic pumps at under $400.
• Can be fabricated by anyone with access to a 3D-printer.
• Is small enough to fit in a drawer when not in use.
• Can be modified and integrated into highly multiplexed platforms.

## 3 Design files

### 3.1 Design files summary

See Table 1.

**Table 1:** Summary of all design files for the FAST Pump.

Design file name	File type	Open source license	Location of the file
FAST Pump.f3d	CAD	GNU GPL v3.	https://osf.io/3r7h4/
Jig.f3d	CAD	GNU GPL v3.	https://osf.io/3r7h4/
Base.stl	STL	GNU GPL v3.	https://osf.io/3r7h4/
Lid 1.2 mm.stl	STL	GNU GPL v3.	https://osf.io/3r7h4/
Lid 1.3 mm.stl	STL	GNU GPL v3.	https://osf.io/3r7h4/
Lid 1.4 mm.stl	STL	GNU GPL v3.	https://osf.io/3r7h4/
Shaft.stl	STL	GNU GPL v3.	https://osf.io/3r7h4/
Jig body.stl	STL	GNU GPL v3.	https://osf.io/3r7h4/
Jig lid.stl	STL	GNU GPL v3.	https://osf.io/3r7h4/
FAST Pump.form	3D-Print	GNU GPL v3.	https://osf.io/3r7h4/
Drive Program.prg	Program	GNU GPL v3.	https://osf.io/3r7h4/

F3D files: These are Autodesk Fusion 360 archive files including all of the parts needed for the pump and jig respectively.

STL files: These are ready to print component files to be imported into any 3D-printing slicer.

FORM file: This is a preconfigured print file for Formlabs Preform software. It can be used to print the entire design, with one click, on a Form 2 or Form 3 3D-printer.

PRG file: Script used to control the stepper motor through the manufacturers software.

## 4 Bill of materials

### 4.1 Bill of materials

See Table 2.

**Table 2:** The complete bill of materials for producing one FAST Pump.

Component	Number	Cost per unit	Cost per unit – $*	Total cost – $*	Source of materials	Material type
Base.stl	1	42.51 DKK **	$6.31	$6.31	–	Formlabs Clear Resin
Lid 1.3 mm.stl	1	19.22 DKK **	$2.85	$2.85	–	Formlabs Clear Resin
Shaft.stl	1	9.81 DKK **	$1.46	$1.46	–	Formlabs Clear Resin
Jig body.stl	1	8.95 DKK **	$1.33	$1.33	–	Formlabs Clear Resin
Jig lid.stl	1	3.60 DKK **	$0.53	$0.53	–	Formlabs Clear Resin
Stepper Motor ARCUS-DMX-J-SA-17	1	£220.5 ***	$286.96	$286.96	https://www.lg-motion.co.uk/products/arcus/	–
USB A to USB Mini B Cable	1	20.78 DKK	$3.08	$3.08	https://dk.rs-online.com/web/p/usb-kabler/1862803/	–
5 mm to 4 mm flexible beam coupling	1	289.42 DKK	$42.96	$42.96	https://dk.rs-online.com/web/p/koblinger-baelgkoblinger/3258195/	Stainless Steel
4x50 mm cylinder pin	6	1.79 DKK	$0.26	$1.56	https://altirustfri.dk/din-7-cylindrisk-stift-4x50-rustfri-a1	Stainless Steel
6x14 mm cylinder pin	1	1.04 DKK	$0.15	$0.15	https://altirustfri.dk/din-7-cylindrisk-stift-6x14-rustfri-a1	Stainless Steel
8x4x3 mm ball bearing	12	6.00 DKK	$0.89	$10.68	https://arduinotech.dk/shop/kuglelejer/ Type: MR84zz	Chrome Steel
16x8x5 mm ball bearing	2	5.00 DKK	$0.74	$1.48	https://arduinotech.dk/shop/kuglelejer/ Type: 688zz	Chrome Steel
Countersunk M4x12 mm bolt	6	2.09 DKK	$0.31	$1.86	https://dk.rs-online.com/web/p/skruer-med-indvendig-6-kant/2328394/	Stainless Steel
Countersunk M3x8 mm bolt	4	1.35 DKK	$0.20	$0.80	https://dk.rs-online.com/web/p/skruer-med-indvendig-6-kant/3044918/	Stainless Steel
M4 nut	6	0.42 DKK	$0.06	$0.36	https://dk.rs-online.com/web/p/sekskantmotrikker/0527252/	Stainless Steel

*Costs in $ converted from DKK, with the exception of the stepper motor, using the current exchange rate at January 21st 2020: 1 DKK = 0,148 USD.

**Costs for 3D-printed parts include support material and are based on a resin price of 1044 DKK/Liter.

***Stepper motor cost converted to $ from £ using the current exchange rate at January 21st 2020: 1 GBP = 1.301 USD.

The only tools needed for assembly of the FAST pump are a set of millimeter hex keys and a hammer. A hard surface, such as a metal plate or table, is also needed.

## 5 Build instructions

### 5.1 3D-Printing

**Fig. 1:**
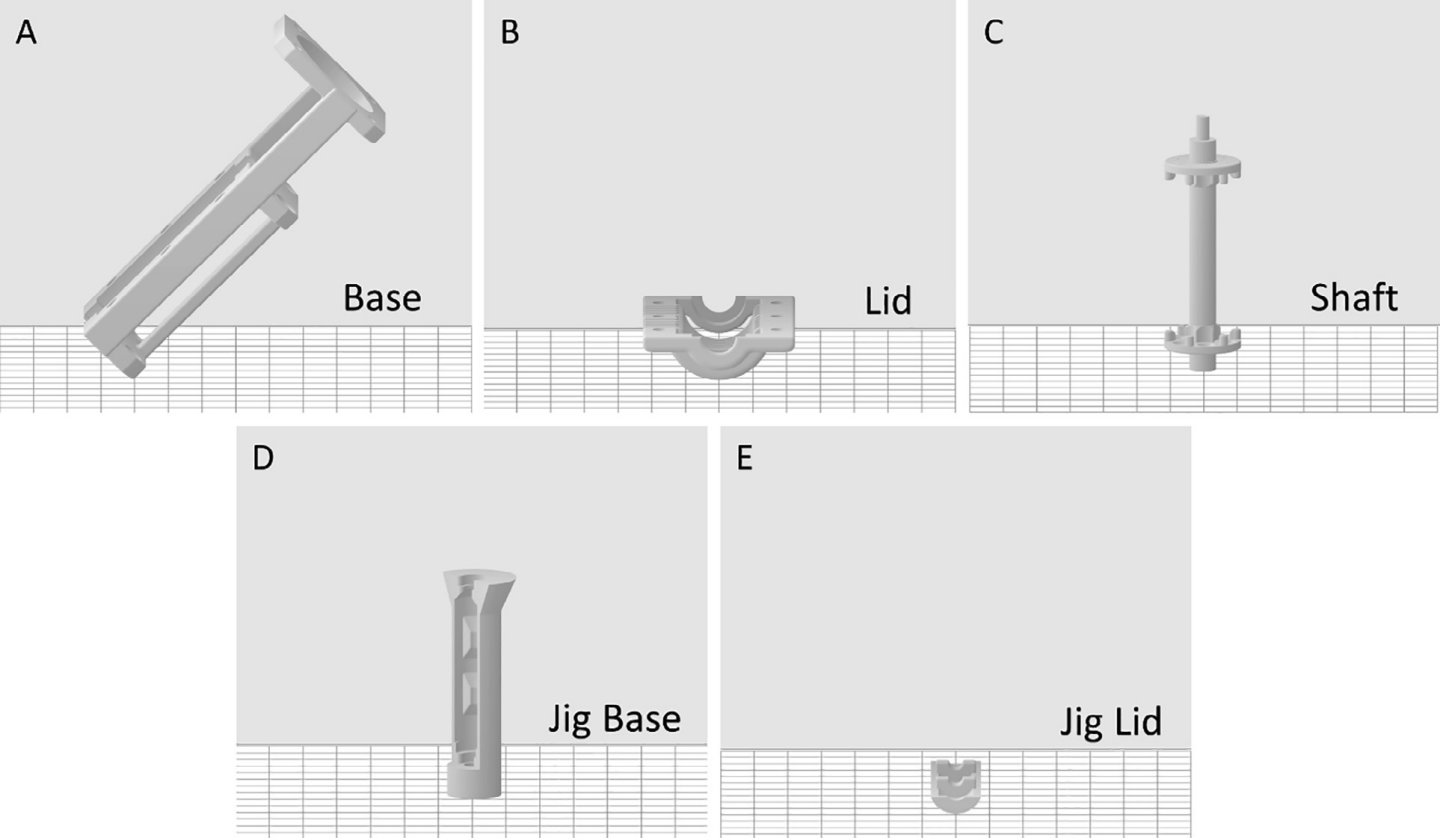
Print orientation for the needed parts. A) Base. Printed at a 45° angle. B) Lid. Printed lying flat up-side-down. C) Shaft. Printed standing. D) Jig Base. Printed standing. E) Jig Lid. Printed lying flat up-side-down.

3D-print all STL part files listed in Table 1 using an appropriate 3D-printer. We recommend using a resin-based (SLA or DLP) printer to ensure the mechanical strength and accurate reproduction of the parts. Specifically; the pump shown in this publication was printed on a Form 2 3D-printer and the included FORM file can be used to directly print it on that machine. The printed parts were post processed in accordance with FormLabs recommendations, i.e. washing in isopropanol using the “Form Wash” washing station (20 min) followed by post curing in the “Form Cure” curing station (60 min, 60 °C). We recommend a layer thickness of 0.05 mm and the part orientations seen in Fig. 1, when using other resin printers.

The default lid used, “Lid 1.3 mm.stl”, has a distance between lid and roller of 1.3 mm and works well for the tubing used in the validation of this pump as discussed later. Included in Table 1 is two additional lid designs with narrower passage, but the lid can be redesigned to fit other dimensions of tubing.

### 5.2 Roller assembly

**Fig. 2:**
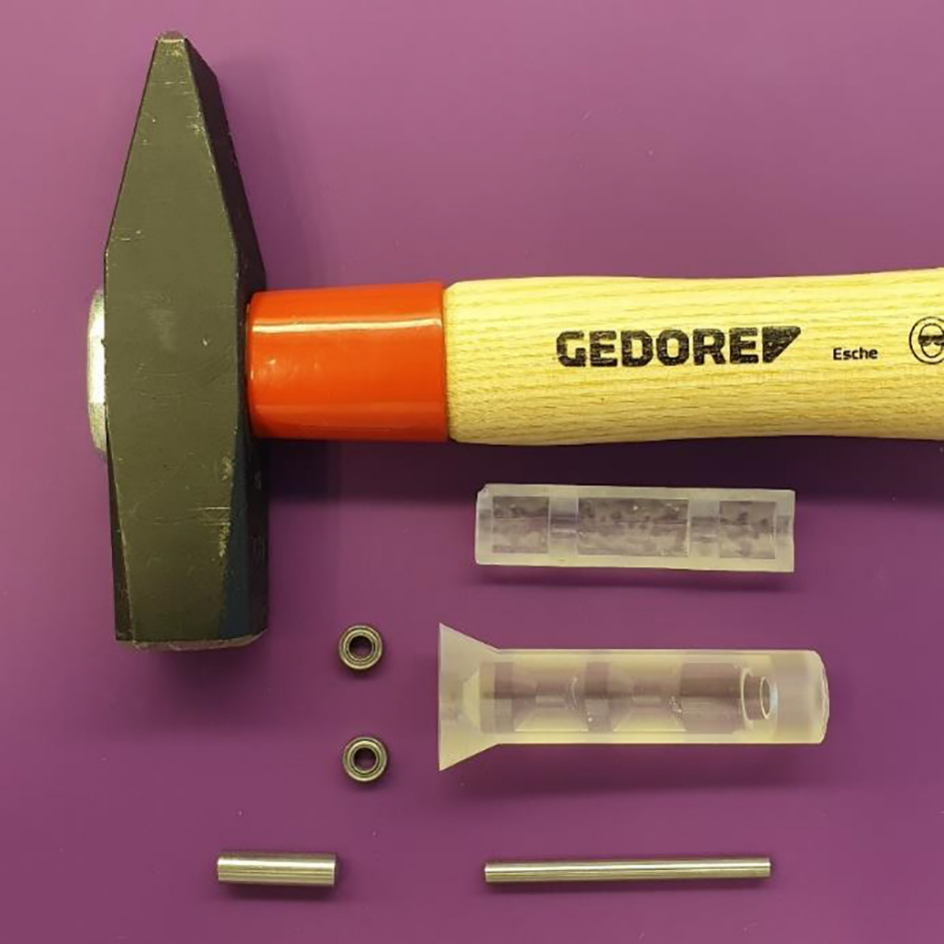
Materials and tools needed for roller assembly.

Assembly of a single pump roller requires the following tools and materials, as seen in Fig. 2.

Tools:

• One “Jig Base”
• One “Jig Lid”
• One 6x14 mm cylinder pin
• A hammer

Materials:

• two 8x4x3 mm ball bearings
• one 4x50 mm cylinder pin

Assembly is done as follows:

1. Insert the ball bearings into the pockets designated in Fig. 3.

**Fig. 3:**
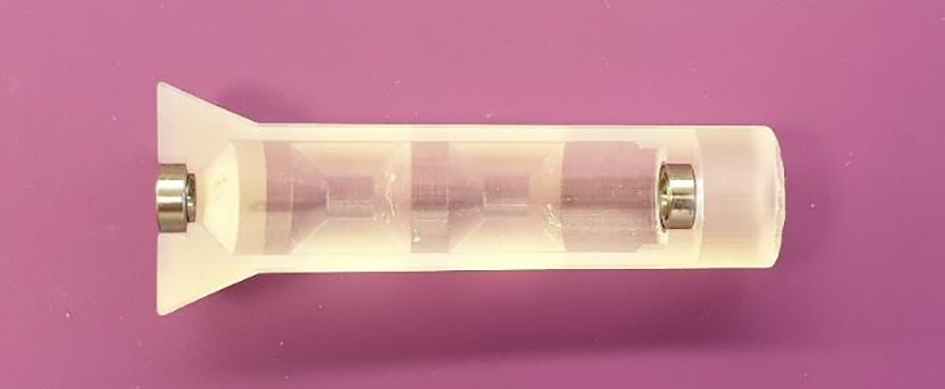
Insert ball bearings into the roller jig.
  *It is important that the bearings lay flat with the bottom of the pockets to ensure that they will be perpendicular to theroller pin.*
2. Place the 4x50 mm cylinder pin into the central trench of the jig.
3. Insert the 6x14 mm cylinder pin into the end of the jig and while pressing down on the roller pin, push against a hard surface, as seen in Fig. 4, until the ball bearing “clicks” in place against the roller pin.

**Fig. 4:**
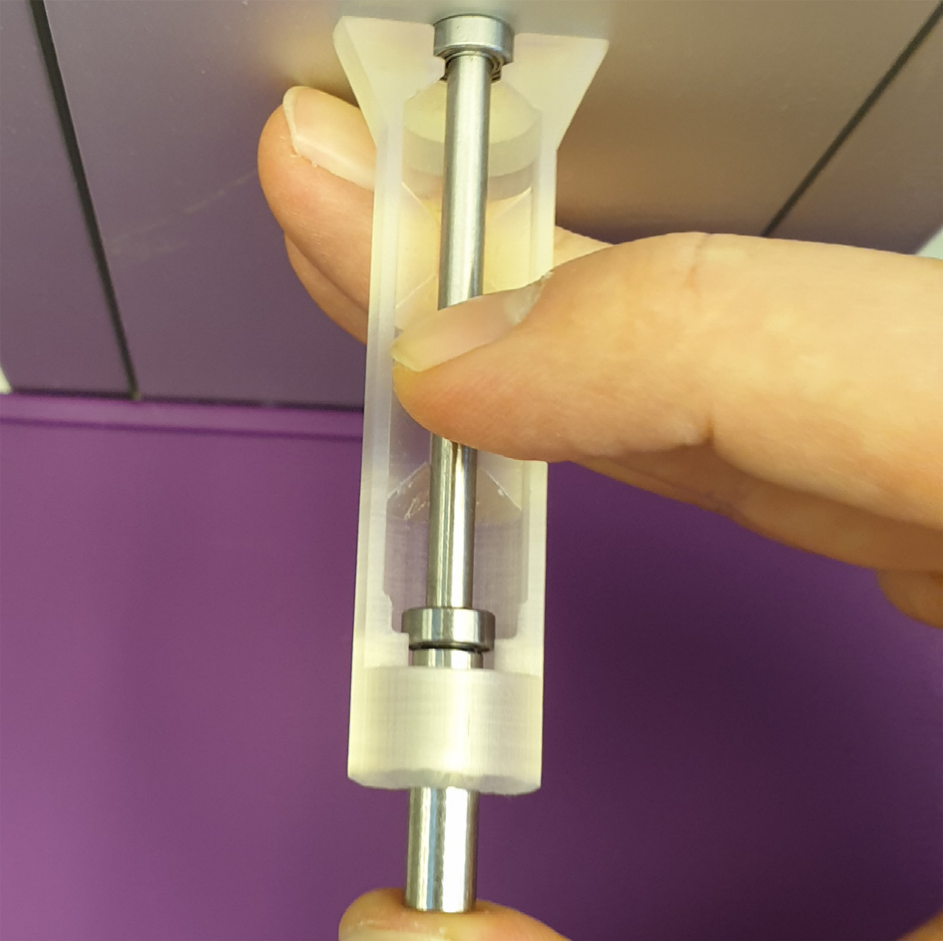
Press the cylinder pin until the roller “clicks” together.
4. Put the jig lid in place and move the jig to a hard horizontal surface, preferably a metal plate, and push it down firmly, as seen in Fig. 5.

**Fig. 5:**
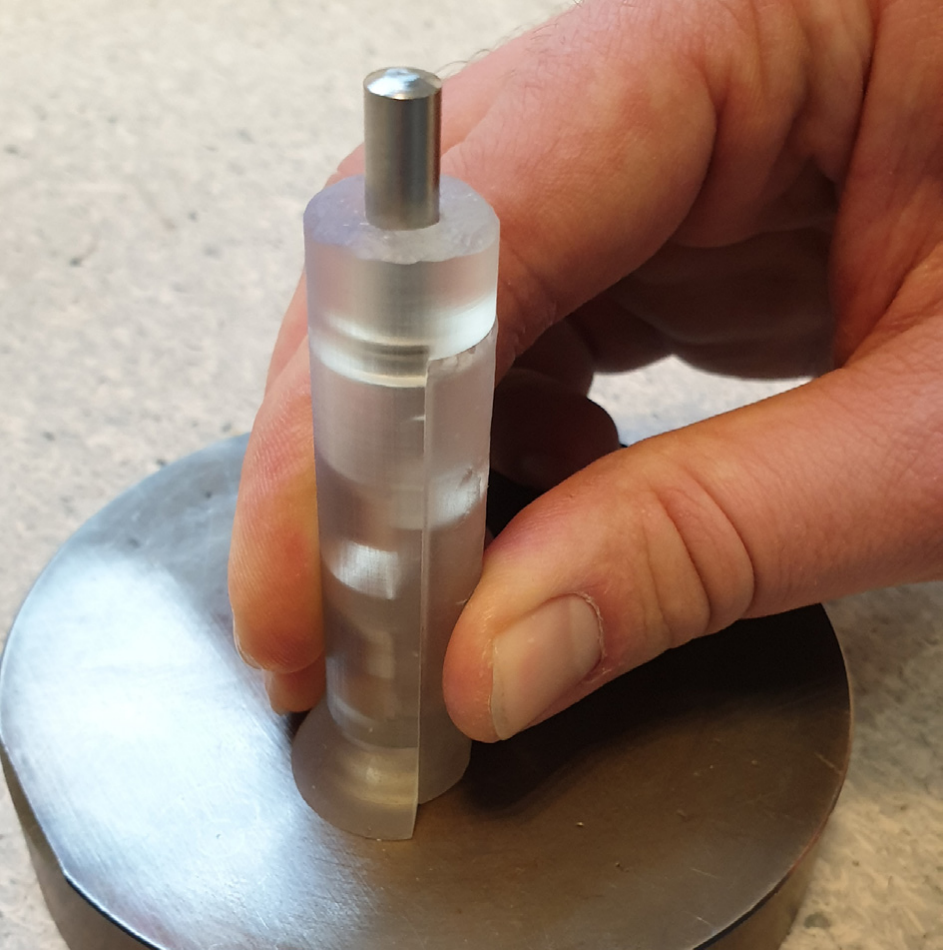
Place the jig on a hard surface and hold it firmly in place.
5. With light but firm taps, drive the metal pin down until you can feel the roller pin hitting the hard surface.
6. Pick up the jig and push on the metal pin to release the finished roller, seen in Fig. 6.

**Fig. 6:**
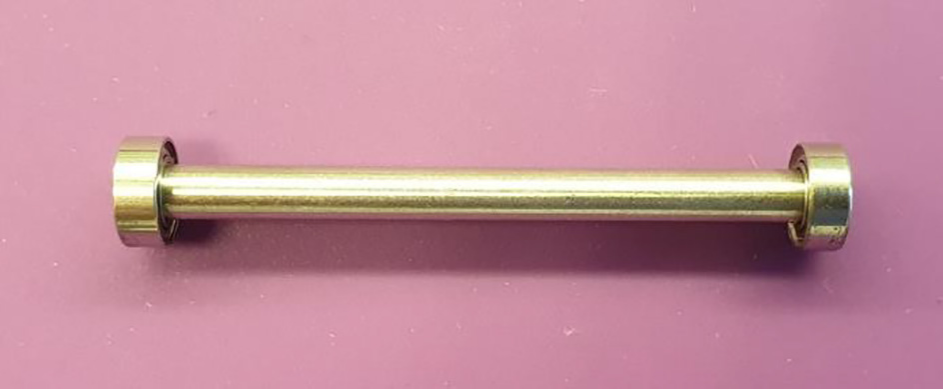
Finished roller.

A total of six rollers are needed for assembly of one FAST Pump. Due to the tolerances of the roller pins and ball bearings some pins cannot be forced into the bearings without issue using the described technique. This can be noticed as a “gravely” feel when the bearing is turned and we recommend that these rollers are not used. Depending on the quality of the pins and bearings, this can be as much as 25–50% of the assembled rollers.

### 5.3 Pump assembly

**Fig. 7:**
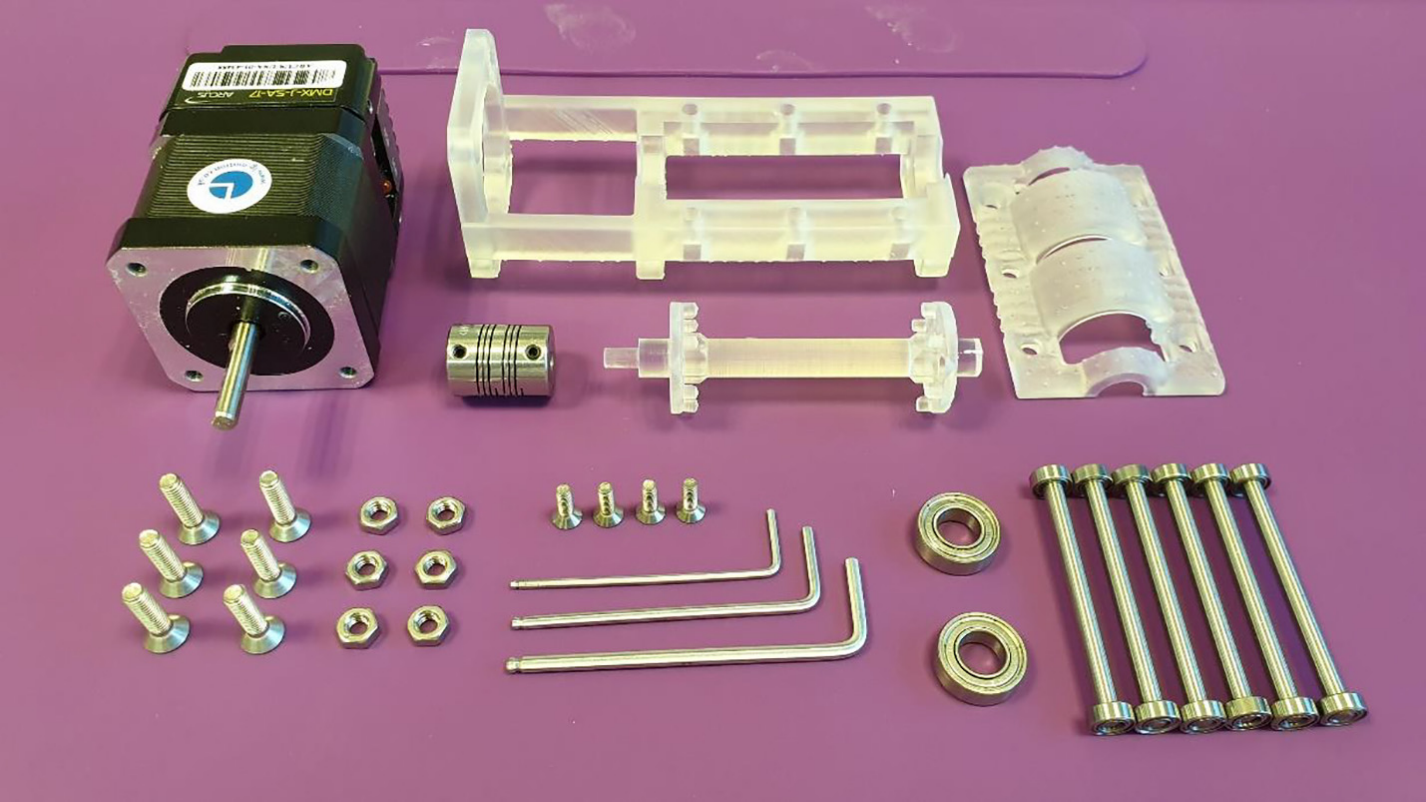
Materials and tools needed for assembly of the FAST Pump.

Assembly of the FAST Pump requires the following tools and materials, as seen in Fig. 7:

Tools:

• 1.5 mm Hex Key
• 2.0 mm Hex Key
• 2.5 mm Hex Key

Materials:

• One “Base”
• One “Lid 1.3 mm”
• One “Shaft”
• Six rollers
• Two 16x8x5 mm ball bearings
• Six countersunk M4x12 mm bolts
• Six M4 nuts
• One stepper motor
• One flexible beam coupling
• Four countersunk M3x8 mm bolts

Assembly is done as follows:

1. Insert the six rollers into their designated pockets on the shaft, as seen in Fig. 8. They should click into place easily.

**Fig. 8:**
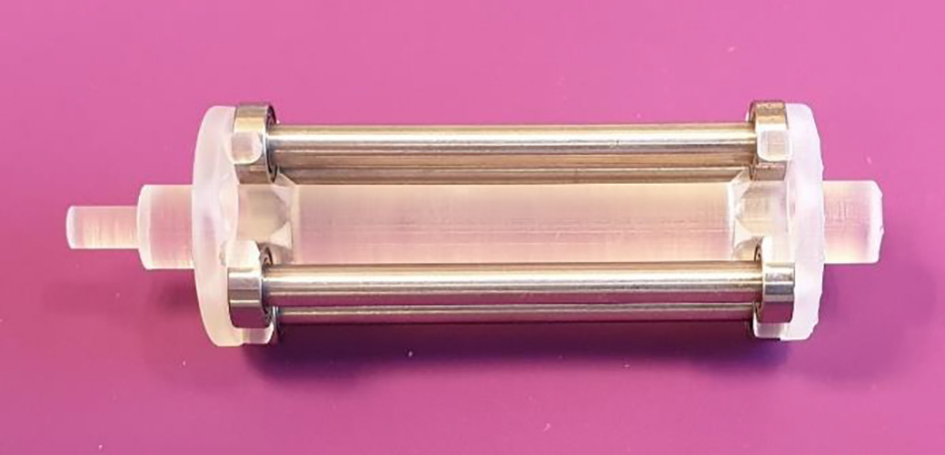
Assemble the roller shaft.
2. Slide on the 16x8x5 mm ball bearings on each end of the shaft and place it in the cradle provided by the base, as seen in Fig. 9.

**Fig. 9:**
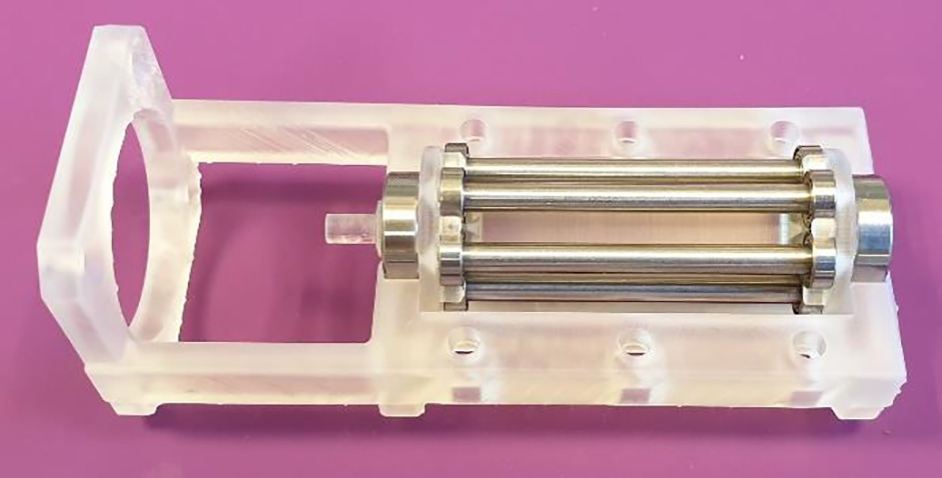
Assemble Roller shaft and Base.
3. Slide on the flexible beam coupling and tighten the locking screw. Make sure to align the screw with the flat cut-out on the shaft and leave a distance of about 1 mm between the coupling and the base, as seen in Fig. 10.

**Fig. 10:**
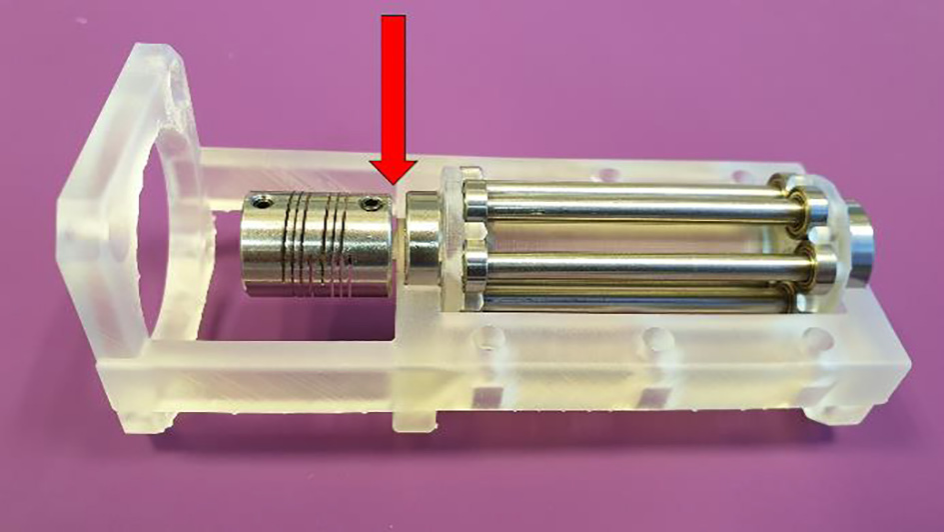
Attach the flexible beam coupling. Arrow denotes a 1 mm gap between coupling and base.
4. Insert the out-going shaft of the stepper motor into the flexible beam coupling and slide the motor against the base. The motor is then secured with the four countersunk M3 bolts, as seen in Fig. 11.

**Fig. 11:**
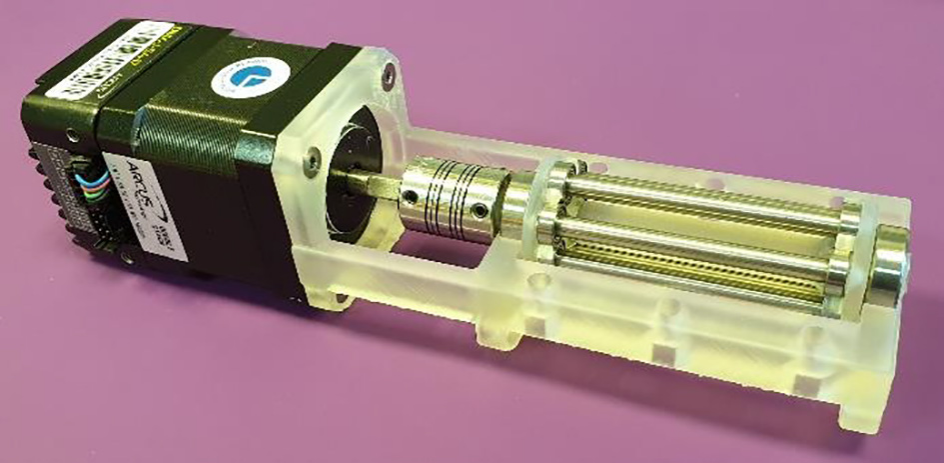
Attach the stepper motor.
5. Align the flat part of the motors out-going shaft with the locking screw by turning the roller shaft, then tighten the locking screw.
6. Place the “Lid 1.3 mm” onto the base, it will align by the pockets for the ball bearings, and turn the entire assembly upside-down.
7. Push an M4 Nut into the designated hexagonal pocket as seen in Fig. 12. The nut does not slide easily into the pocket, this is by design, and it only needs to stay in place.

**Fig. 12:**
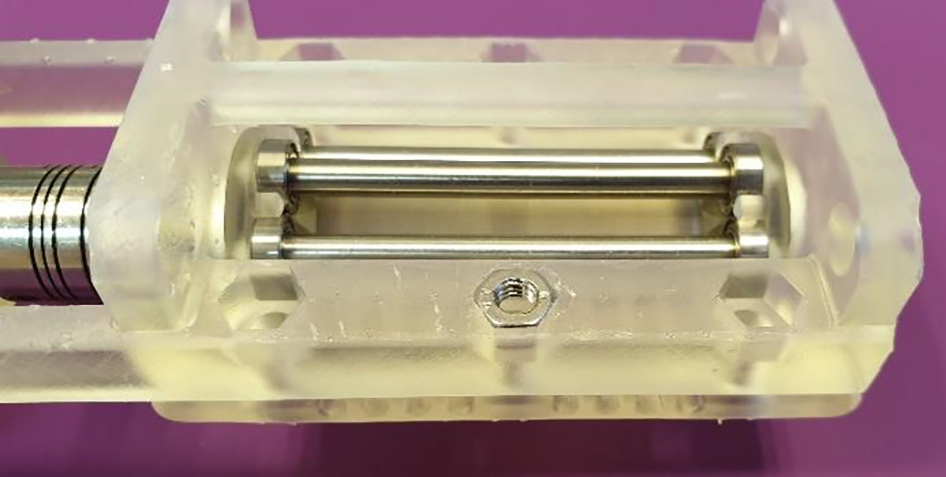
Insert M4 Nuts into the underside of the base.
8. Insert a countersunk M4 bolt from the top of the assembly and thread it into the nut.
9. Repeat step 7 and 8 for the other five holes.
10. Gradually tighten the bolts. As the bolts are tightened the nuts will be forced into the pockets, as seen in Fig. 13. Tighten until you can feel that the nuts have reached the bottom of the pockets.

**Fig. 13:**
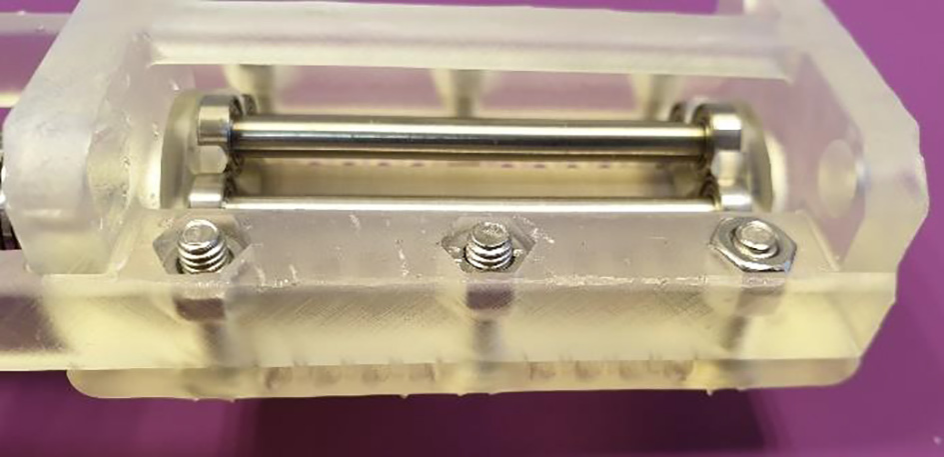
Tighten the bolts to seat the nuts.
11. The FAST Pump is now assembled and ready to be used, as seen in Fig. 14. Addition of tubing is covered in section 6, below.

**Fig. 14:**
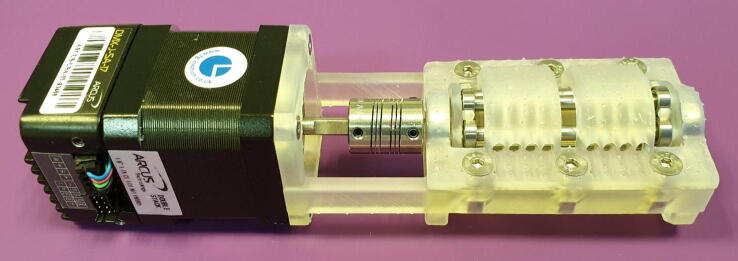
Assembled FAST Pump.

## 6 Operation instructions

**Fig. 15:**
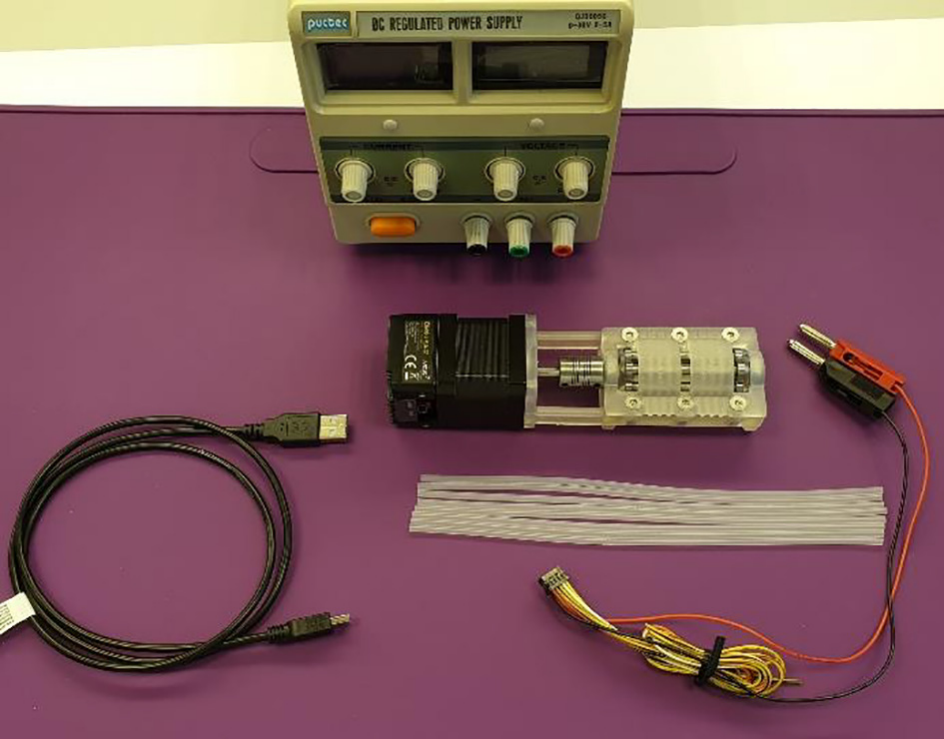
Materials needed to operate the FAST Pump.

Operation of the FAST Pump requires the following materials, as seen in Fig. 15:

• Pieces of tubing.
  *This can be any tubing ≤3 mm in outer diameter with a wall thickness of ~1 mm. Tubing <3 mm in outer diameter need to have stops in order to be used. For tubing with slightly thinner or thicker walls than 1 mm the “Lid 1.2 mm” or “Lid 1.4 mm” can be used respectively. For other tubing dimensions the lid design needs to be modified, this can be easily done in the provided “FAST Pump.archive” file.*
• One USB A to USB Mini B Cable
• A lab-scale power supply set to 24 V DC-power.
  *This is simply to provide power for the stepper motor. The motor can instead be fitted with a 24 V, 2.5A, DC plug-in power supply for easier use.*

The pump is operated as follows, assuming a computer running Microsoft Windows:

*Before first time operation, install drivers and control software for the stepper motor. They can be found at* *https://www.arcus-technology.com/products/integrated-stepper-motors/nema-17-integrated-usb-stepper-basic/* *under the software tab.*

1. Insert tubing into the Lid as shown in Fig. 16.

**Fig. 16:**
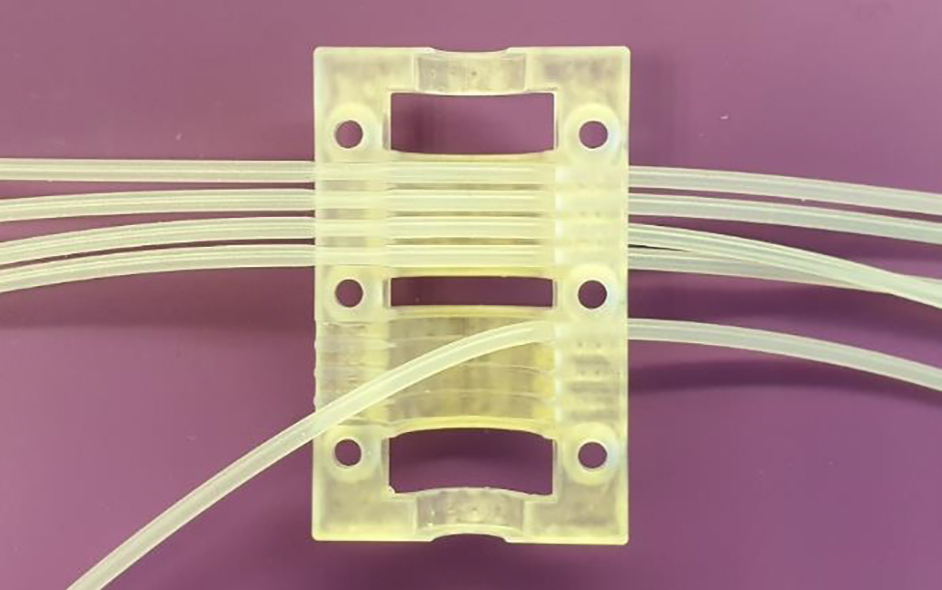
Mounting of tubes in the pump Lid.
2. Fasted the Lid and make sure that it is positioned correctly according to Fig. 17.

**Fig. 17:**
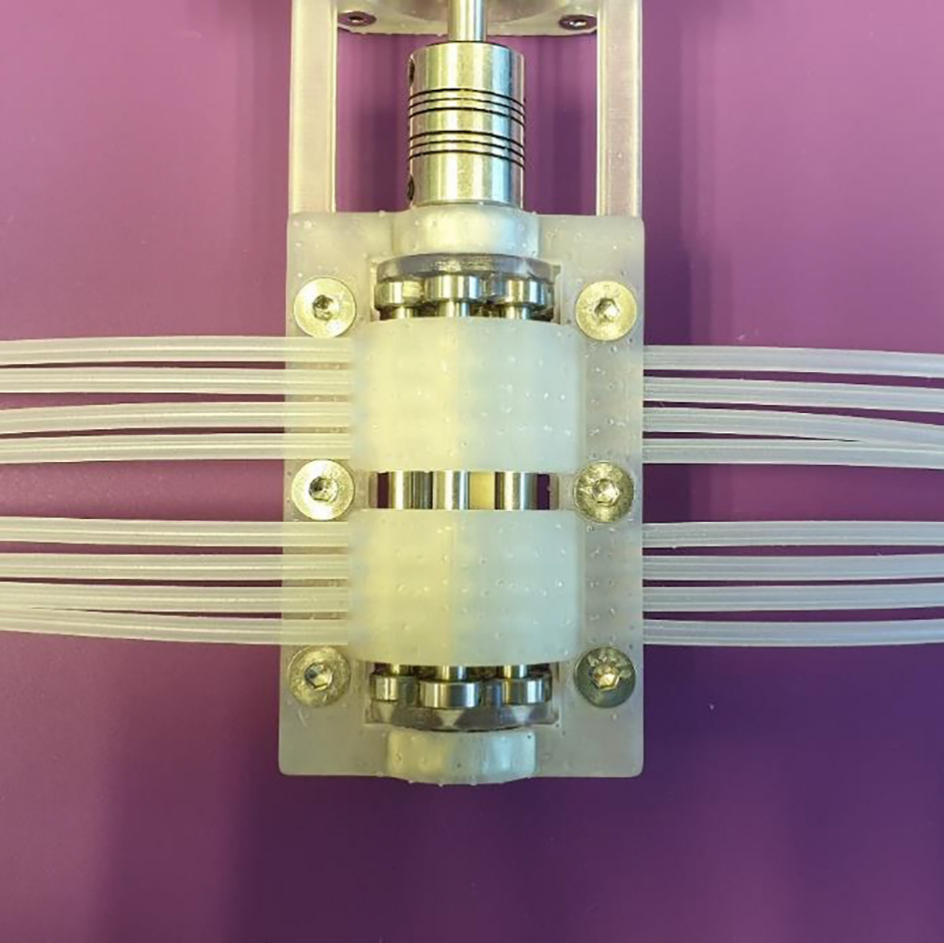
Correct orientation of the lid.
*The lid design makes the pump unidirectional and incorrect positioning of the lid will result in pump failure and potential damage to the pump.*
3. Attach the USB cable and the power cable, as seen in Fig. 18.

**Fig. 18:**
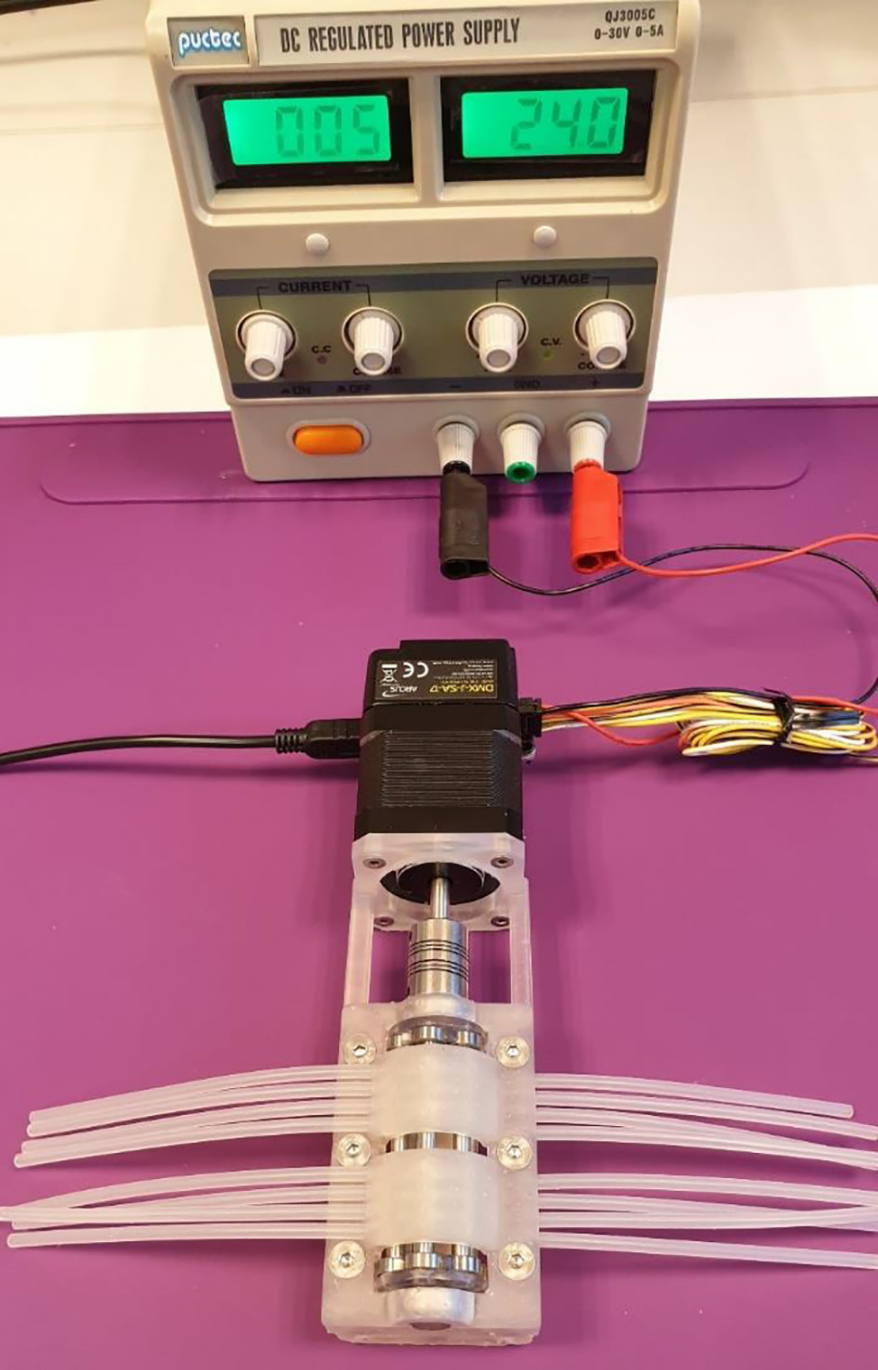
Complete pump setup.
*If using a power supply, make sure to turn it on before connecting the pumps power cable.*
4. Run ”SOFT-EXE-DMX-J-SA-123″, found in the now added “Arcus Technology” folder in the start menu.
5. Verify the Run Current, as shown in Fig. 19, according to Table 4 depending on the tubing used. When changes have been made, remember to press “Download”.

**Fig. 19:**
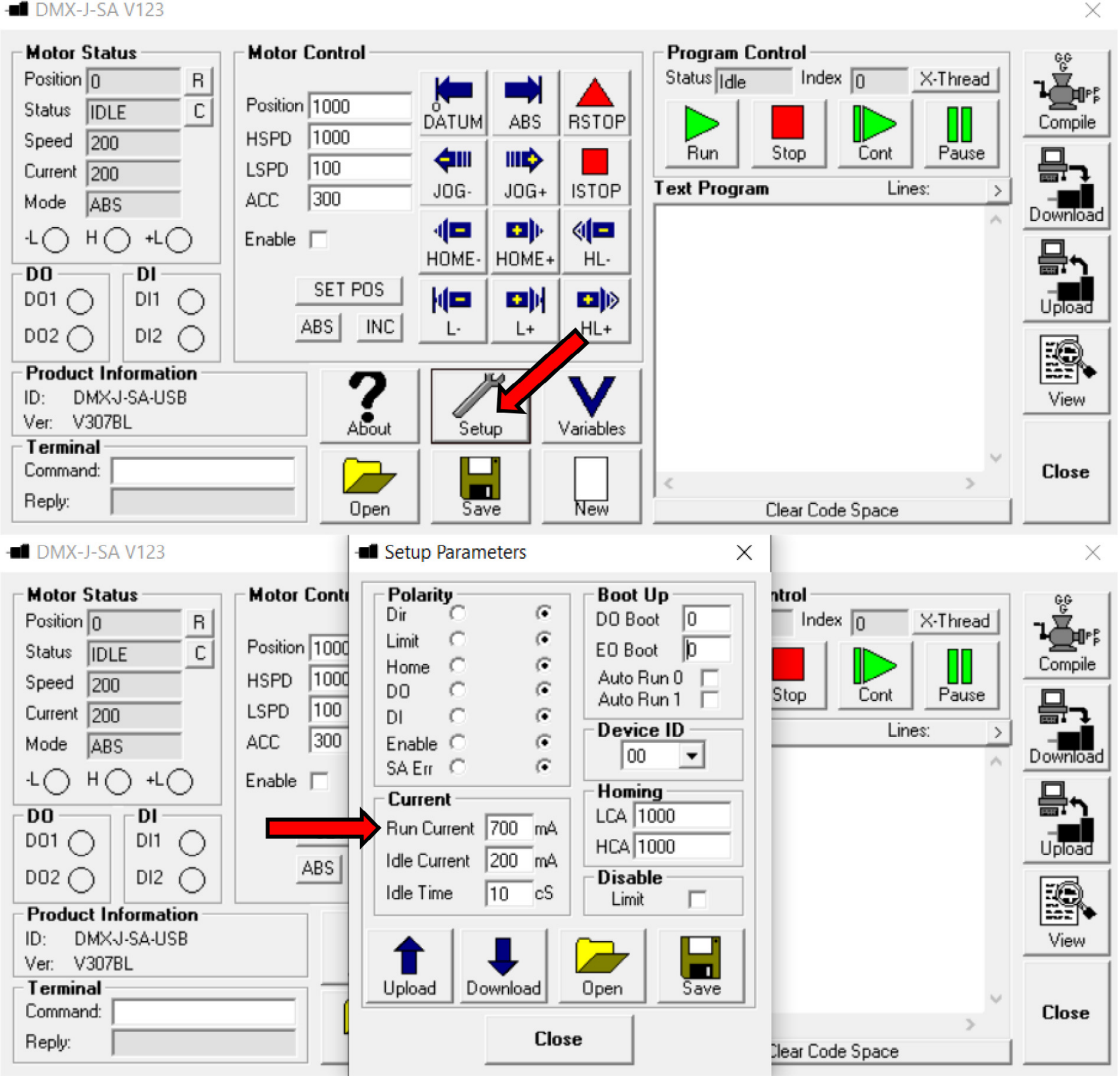
Verification of Run Current. Found under “Setup”.
6. Load “Drive Program.prg” by pressing “Open” and browsing to the file location.
7. Motor Speed, in “Pulses/s”, and Pumping Time, in minutes, can be set by changing the designated lines of code in Fig. 20. When changes have been made press “Compile” followed by “Download”.

**Fig. 20:**
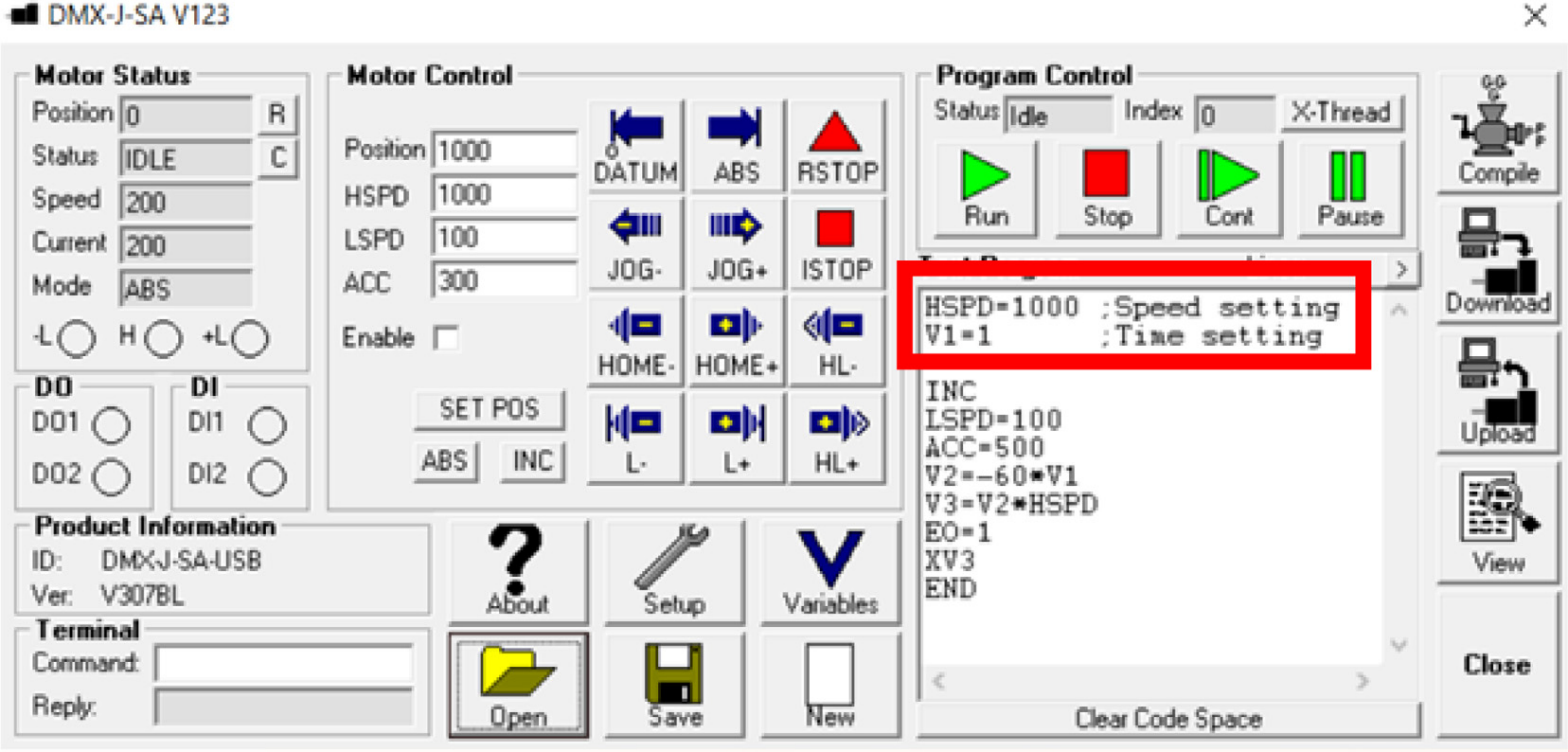
Setting Motor Speed and Pumping Time.
8. Press “Run” to start the pump.
*The first time the pump is started, after the lid has been attached, it is sometimes necessary to give the shaft a nudge to get it going. If the program needs to be aborted early it is recommended to “RSTOP” button as this allows the motor to decelerate. The “Stop” button can also be used but stops the motor immediately, putting more stress on all parts.*

## 7 Validation and characterization

### 7.1 Tubing considerations

The FAST Pump has been tested with low cost silicone tubing that can be easily purchased in bulk. Two different types of tubing commonly used for peristaltic pumps have also been tested. Both are of two-stop configuration with 152 mm between the stops. When cut in the middle this produces two pieces of tubing for the FAST Pump with a single stop to be seated at the inlet side of the lid. The different types are listed in Table 3.

**Table 3:** Tubing used for validation of the FAST Pump.

Type	Comment	Cost per package – DKK	Cost per pump – DKK	Cost per pump – $*	Source of materials
Pharmed BPT	2.05 mm OD, sold in packages of 6.	324.00 DKK	216.00 DKK	$32.06	https://dk.vwr.com/store/catalog/product.jsp?catalog_number=224-0550
0.25 mm ID					
Tygon™ LMT 55	2.31 mm OD, sold in packages of 12.	219.75 DKK	73.25 DKK	$10.87	https://dk.vwr.com/store/catalog/product.jsp?catalog_number=VERN070534-05-ND
0.51 mm ID					
Silicone	3.00 mm OD, sold in package of 25 m.	167.20 DKK	10.20 DKK**	$1.51	https://dk.vwr.com/store/catalog/product.jsp?catalog_number=DENE3100103/25
1.00 mm ID					

*Costs in $ converted from DKK, using the current exchange rate at January 21st 2020: 1 DKK = 0,148 USD.

**Assuming length of tubing used for each channel equivalent to the two other types, 190.5 mm.

**Table 4:** Run currents needed to operate the FAST Pump depending on Lid and tubing used.

		Lid-to-Roller distance [mm]		
		1.2	1.3	1.4
Tube ID [mm]	0.25	600 mA*	500 mA*	400 mA**
	0.51	1200 mA*	1000 mA**	800 mA***
	1.00	700 mA*	700 mA*	600 mA**

* Denotes fully functional pumping.

** Denotes partly functional pumping with some channels not pumping.

*** Denotes Non-functional pumping. All currents listed are valid for motor speeds up to 10,000 pulses/s.

Depending on Lid and tubing used, different torque output is needed from the stepper motor, this is represented by the Run Current that can be set in the control software. As can be seen in Table 4, Tygon™ especially is harder to compress and puts considerably more strain on the motor than Pharmed BPT and silicone. As Run Current is also directly correlated to the amount of heat the motor produces, we recommend that Tygon™ is avoided when possible.

### 7.2 Pump performance

**Fig. 21:**
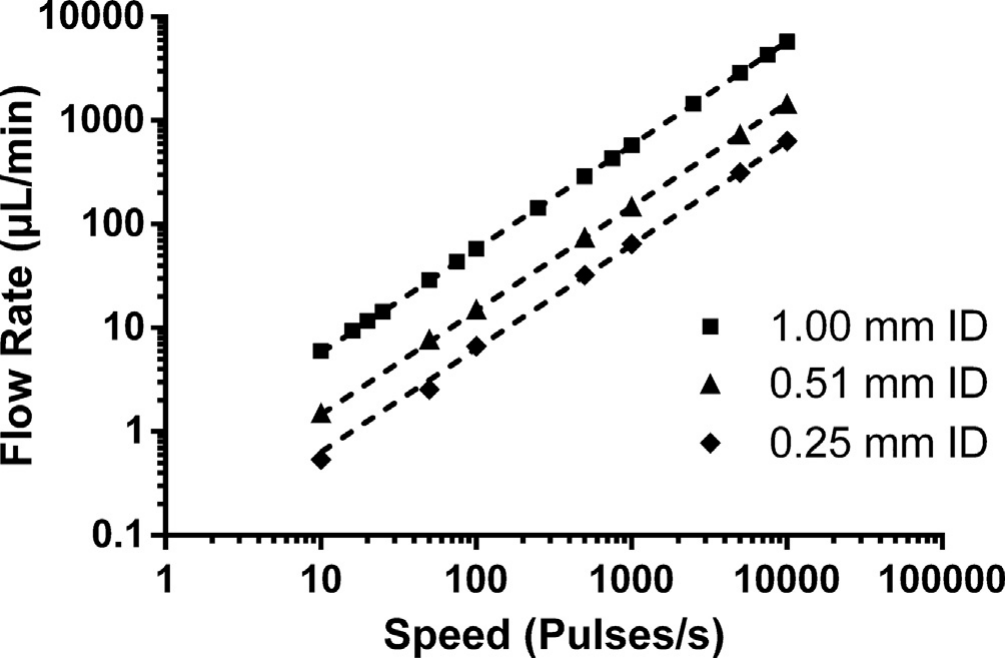
Flow rate as function of motor speed for the three types of tubing. Each data point in the average of all channels (n = 8). The equations and R^2^ values for the linear fits are listed below. Each fit has been forced through 0,0. Error bars representing standard deviation are not depicted as they are smaller than the individual points of the plot. Equations for the linear fits: 1.00 mm ID: Y = 0.5750*X, R^2^ = 0.9994 | 0.51 mm ID: Y = 0.1468*X, R^2^ = 0.9973 | 0.25 mm ID: Y = 0.0632*X, R^2^ = 0.9998.

**Fig. 22:**
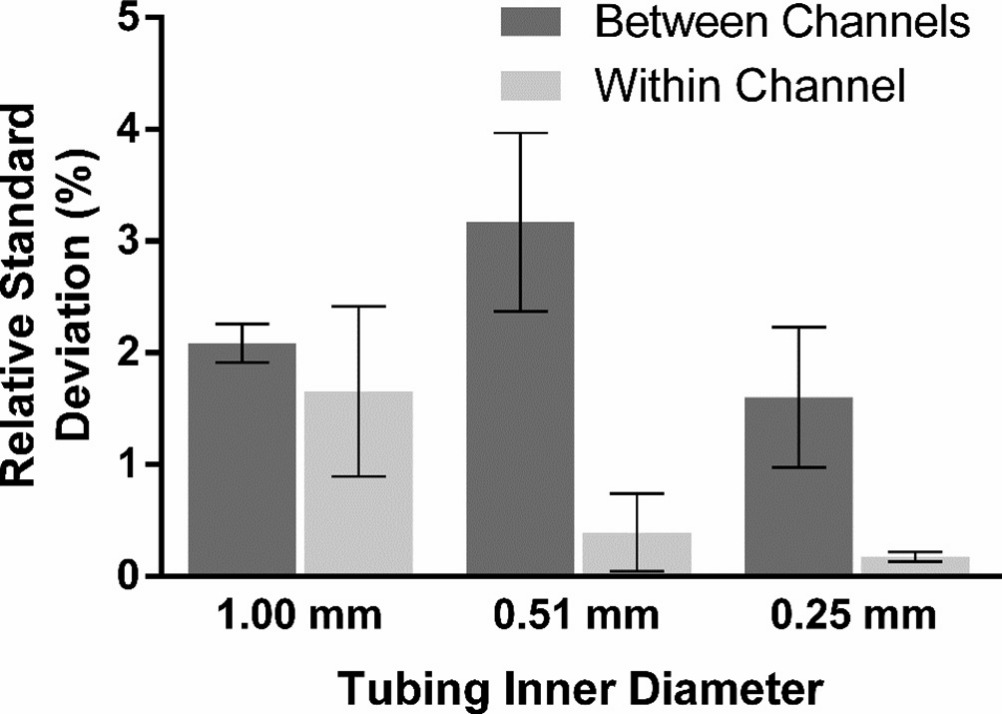
Relative standard deviations for the different tubing sizes. Dark grey denotes the standard deviation between the flow channels at each flow rate (n = 8). Light grey denotes the standard deviations within channels across the entire flow rate range (n = 7).

**Fig. 23:**
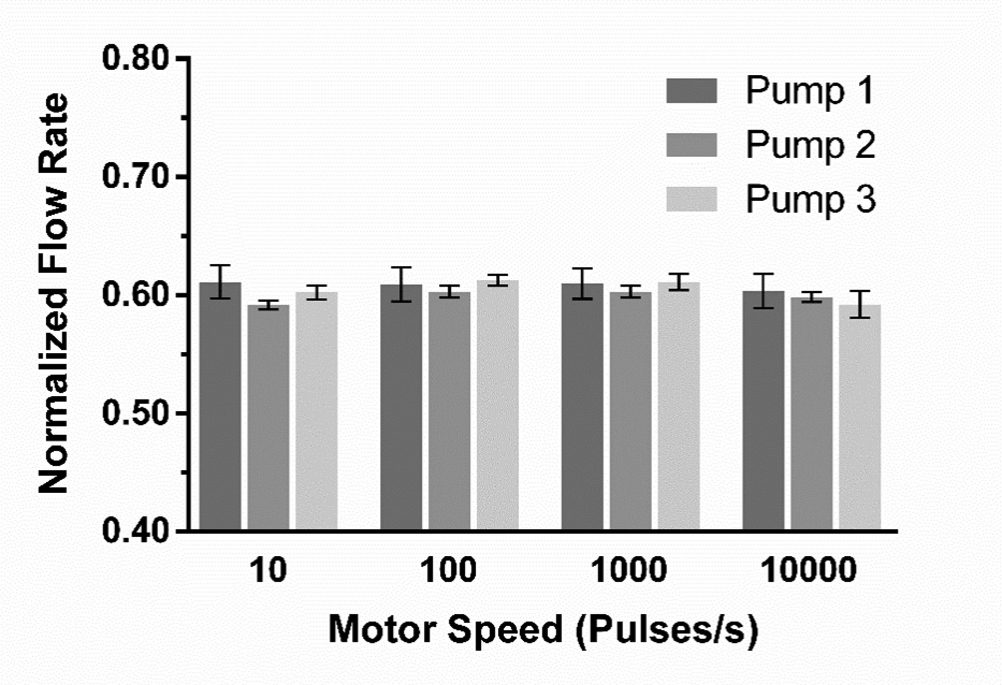
Comparison of the flow rates of three iterations of the FAST Pump (n = 8 for each pump). Normalized flow rate is flow rate divided by motor speed.

Pumping was performed in the range of 10–10000 pulses/s, which corresponds to RPMs of 0.1875–187.5. While the motor will accept values lower than 10, no change in speed can be seen. This is due to the way the motor controller works and sets the lower speed limit. The motor can achieve much higher speeds than 10,000 pulses/s, with the given maximum being 200,000 pulses/s, but we have chosen to limit our characterization to a range that is more representative of the intended use of the pump. Flow rate was measured by pumping water for a set amount of time, collecting the flow-through and weighing it on an analytical balance (Mettler Toledo AE100 Analytical Balance, Mettler Toledo, Zaventem, BE). For motor speeds 51–1000 pulses/s at least 500 µL was collected in a 1.5 mL Eppendorf tube and for motor speeds 1001–10000 pulses/s at least 5 mL was collected in a 15 mL Falcon tube. For motor speeds ≤50 pulses/s a commercial flow meter (Mitos Flow Rate Sensor, Dolomite Microfluidics, Royston, UK) was used to measure flow rate, this because of the long operation needed to collect the necessary volume of water for weighing. As seen in Fig. 21, the relationship between motor speed and output flow rate show good linearity over the entire studied range. The relative standard deviations for each of the three types of tubing can be seen in Fig. 22. The relative standard deviation for each channel across the full range can be seen as measure of the pumps precision and is mainly affected by the linearity of the motors speed curve and the compliance of the tubing. As expected, these are low for the commercial pump tubing at under 1%. For the silicone tubing it is slightly higher, most likely due to the compliance of the soft material. The relative standard deviation between the channels is mainly affected by the fit between parts, and thus the 3D-print quality. As expected this is higher than the per channel deviation, but still low at approximately 2% for the 1.00 mm ID and 0.25 mm ID tubing, both using the “Lid 1.3 mm”. For the 0.51 mm ID tubing, using “Lid 1.2 mm”, the deviation is slightly higher most likely as an effect of the hardness of the tubing. Reproducibility of the pump manufacturing was investigated by comparing the normalized flow rates of three pumps across the entire flow rate range. As can be seen in Fig. 23 the fabrication process is highly reproducible with pump-to-pump standard deviation at less than 1% for all flow rates.

To put the FAST Pump in context it can be related to comparable systems, commercial or found in literature. An example is a commercially available pumping system, Takasago 6-channel pump (https://www.takasago-fluidics.com/products/6-channel-pump-peristaltic-pump), which has a similar form factor. This pump claims a flow rate accuracy between channels of ± 10% using 1 mm ID, 2 mm OD, silicone tubing. Another example is the 3D-printed pump presented by Alam et al. [34], with an R^2^ of 0.9975 for flow rates in the range of 40–230 µL/min with 0.8 mm ID, 1.8 mm OD silicone tubing. This is comparable linearity to the FAST Pump, but over a much smaller range and with only a single flow channel. A last example is the 3D-printed pump presented by Vaut et al. [29]. This single channel pump is shown to operate with an R^2^ of 0.9998 for flow rates in the range of 4 – 63 mL/min using 4 mm ID, 7.2 mm OD silicone tubing. This is again comparable linearity to the FAST Pump, but using significantly larger bore tubing and only a single flow channel.

### 7.3 Relevant use case – multiplexed, digital, ELISA

**Fig. 24:**
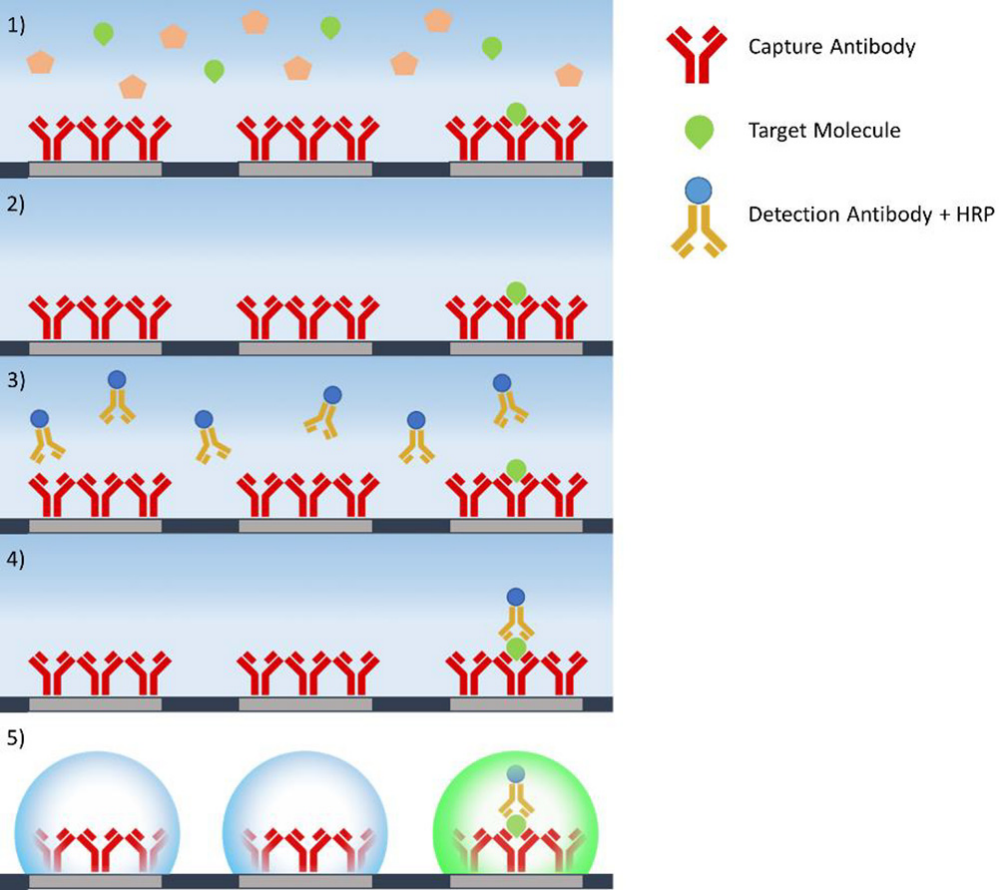
Workflow for a digital sandwich ELISA assay on MDA. **1)** Sample incubation with antibodies immobilized on hydrophilic spots: only one target molecule (green) statistically binds to a spot. **2)** Wash. **3)** Incubation with detection antibodies (yellow) conjugated with HRP. **4)** Wash. **5)** Production of droplets with enzyme substrate and count of the number of spots using a fluorescence microscope. (For interpretation of the references to color in this figure legend, the reader is referred to the web version of this article.)

The digital Enzyme Linked ImmunoSorbent Assay (ELISA) of recombinant Tau protein is carried out on a MicroDroplet Array (MDA) with water/air interface previously developed in our group [13], where droplets are generated on a glass slide patterned with hydrophilic spots surrounded by a hydrophobic background. The basic principle of MDA-ELISA can be seen in Fig. 24. The sandwich assay has a capture antibody attached on the surface that binds to the target molecule and a detection antibody conjugated with Horse radish peroxidase (HRP). A fluorogenic enzyme substrate is used for readout.

**Fig. 25:**
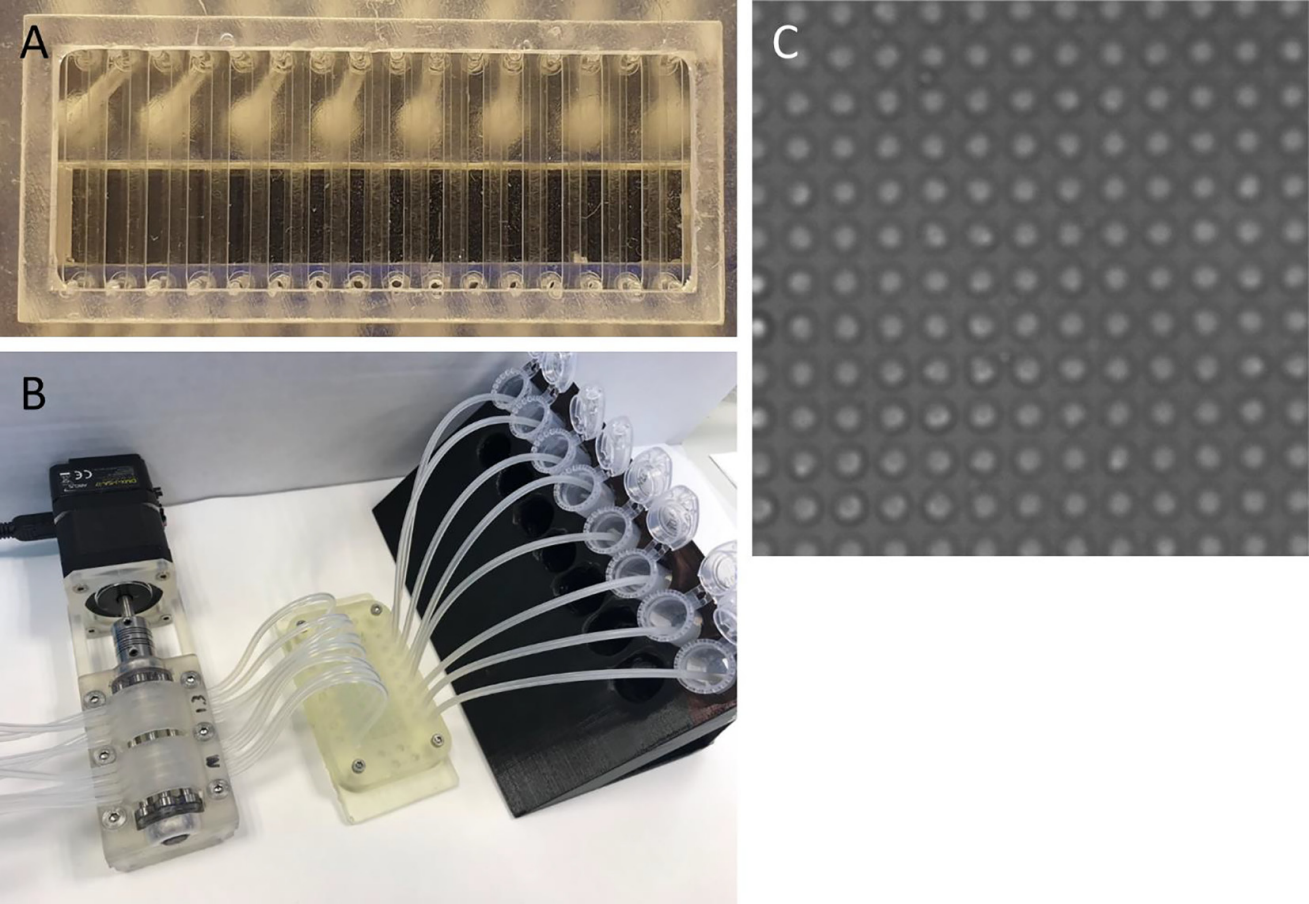
The fluidic system used for MDA-ELISA. A) Channels formed by the PDMS gasket. B) Complete fluidic system including FAST Pump and Inlet reservoirs. C) MDA with formed droplets.

The patterned glass slide is placed in a 3D printed adapter together with a poly-dimethyl-siloxane (PDMS) gasket. When pressed on top of the slide the gasket forms 16 channels, as seen in Fig. 25A, which are connected to ports in the 3D-printed adapter. The inlet ports allow for tubing to be connected between the adapter and liquid reservoirs, whereas the outlet ports are connected to the FAST Pump, which provides negative pressure to induce flow. The assembled system can be seen in Fig. 25B. The passage of liquid through the channels generates femtoliter sized droplets on the hydrophilic spots of the patterned glass slide. Slow and consistent pumping is critical for droplet formation and the FAST pump produces good quality droplet arrays, comparable to those produced by a commercial peristaltic pump (205U Casette pump, Watson-Marlow, Falmouth, JM). An example of well-formed droplets can be seen in Fig. 25C, this uniform pattern is fundamental for the successful performance of the assay. To carry out the assay, phosphate buffered saline (PBS) solutions containing capture antibody, tau protein and detection antibody are sequentially flushed through the channels. In order to functionalize the hydrophilic spots of the microdroplet array, a solution of the capture antibody (100 nM) is loaded into the channels and statically incubated for 1 h, serving as the base for ELISA. Since this kind of immunoassay is diffusion-limited flow rates in the order of 1 – 10 µL/min are necessary to give the biomolecules enough time to reach the surface and form immunocomplexes (Fig. 24:1). Therefore, 3 mL of solutions containing different concentrations of Tau protein (100fM − 10aM) are slowly perfused through the respective channels, with a nominal flowrate of 6 µL/min, corresponding to a total incubation time of about 8 h (Fig. 24:1). After a wash (Fig. 24:2), the detection antibody, conjugated with HRP (10 pM), is perfused for 8 h (Fig. 24:3). After a second wash (Fig. 24:4), 20 µL of a fluorogenic enzyme substrate solution, containing 200 µM ampliflu red and 18 mM hydrogen peroxide, is flushed through each channel, at 6 µL/min, leaving microdroplets on the hydrophilic spots (Fig. 24:5). Upon incubation, the enzyme conjugated to the detection antibody generates a strong fluorescent signal that can be detected by a fluorescent microscope.

**Fig. 26:**
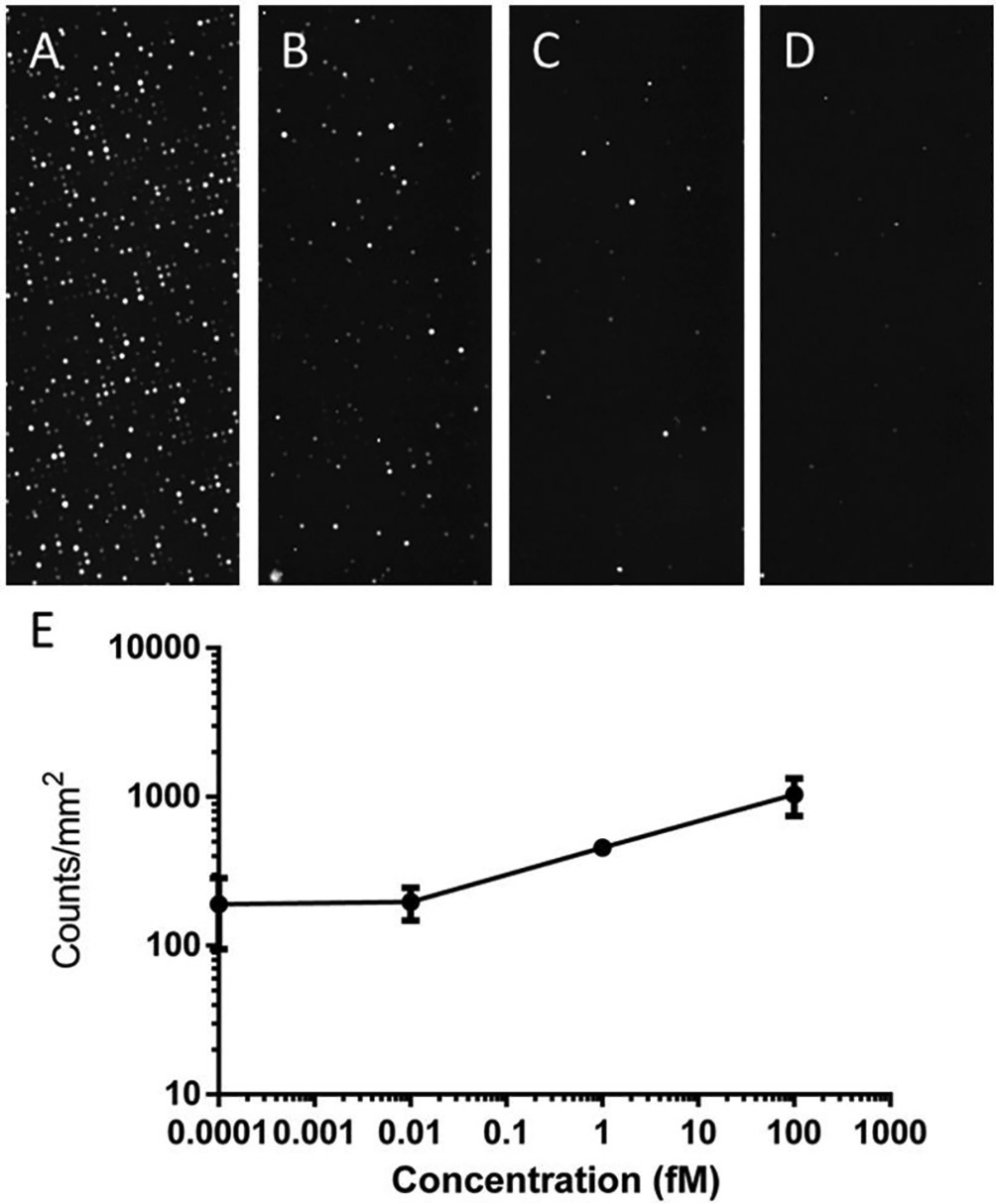
Results of the Single enzyme-linked immunosorbent assay for the detection of Tau protein. A)-D) show representative fluorescence micrographs of a microdroplet array (MDA) functionalized for the immobilization and quantification of Tau protein. **A)** 100 fM. **B)** 1 fM. **C)** 10 aM. **D)** Negative control. The MDA is imaged on a Zeiss Axio ObserverZ1 microscope equipped with a Zeiss Axiocam MRm B/W camera using 20x magnification. Excitation wavelength is 555 nm and emission wavelength is 583 nm. The number of fluorescent droplets, taken as a measurement of the concentration of the target analyte in the sample, is counted using pattern recognition in the software ImageJ after binarizing the signal from the spots with an intensity threshold applied to all the fluorescence micrographs. **E)** Standard curve from quantification of the number of spots in the MDA for each concentration (n = 2).

Due to the low concentrations of Tau protein used, the femtoliter sized droplets will statistically contain a single analyte molecule or no analyte at all. Hence, only droplets containing analyte molecules will generate fluorescence. The results of the assay can be seen in Fig. 26, where a linear relationships is observed between the flushed concentration of Tau and the number of spots. As transfer of Tau to the MDA is dependent on flow rate, slow and consistent pumping is necessary. The linear relationship from 10 fM indicates excellent pumping consistency.

The small footprint of the FAST pump allows for it to be easily placed inside an incubator for temperature-controlled experiments. Larger pumps are difficult to fit into incubators, instead relying on the usage of long tubing with considerable dead volumes as a result. A comparison between the commercial pump and the FAST Pump can be seen in Table 5. The two most striking differences between the commercial pump and the FAST Pump is the price and the size. Depending on retailer the Watson-Marlow 205U can cost anywhere from $6200 to $9000, which is at least 17x the cost of fabricating the FAST Pump. As can be seen in Fig. 25A the 3D-printed adapter allows for 16 parallel experiments to be run, but as this would require two 8-channel pumps (or one 16 channel pump) the price of commercial equipment is a hindrance. The large footprint of commercial systems also result in a considerable amount of bench space having to be reserved for each pump. This will be even more critical if the assays, and hence the system, is placed in an incubator for temperature control. As such, this utilization of the FAST Pump showcases some of the disruptive properties of the system: It is cheap and compact alternative to commercial peristaltic pumps and can be fabricated by anyone with minimum training in 3D-printing.

**Table 5:** Comparison between a typical, commercial, 8-channel peristaltic pump and the FAST Pump. Listed flow rate ranges assume tubing IDs of 0.25–1.0 mm. Data for commercial pump gathered from manufacturer.

	FAST Pump	Watson-Marlow 205U
Number of channels	8	8
Flow rate range	0.7–5750 µL/min (tested)	1.3 – 3760 µL/min
Dimensions (L × W × H)	168 × 45 × 42 mm	334 × 148 × 161 mm
Weight	0.5 kg	7.6 kg
Control	USB	Keypad or analogue remote
Price	$362	>$6000

## CRediT authorship contribution statement

**Alexander Jönsson:** Conceptualization, Methodology, Investigation, Data curation, Writing - original draft, Visualization. **Arianna Toppi:** Investigation, Visualization. **Martin Dufva:** Conceptualization, Writing - review & editing, Supervision, Project administration, Funding acquisition.

## Declaration of Competing Interest

The authors declare that they have no known competing financial interests or personal relationships that could have appeared to influence the work reported in this paper.
